# Lifetime history of indoor tanning in young people: a retrospective assessment of initiation, persistence, and correlates

**DOI:** 10.1186/1471-2458-12-118

**Published:** 2012-02-10

**Authors:** Karen Lostritto, Leah M Ferrucci, Brenda Cartmel, David J Leffell, Annette M Molinaro, Allen E Bale, Susan T Mayne

**Affiliations:** 1 Yale School of Public Health, 60 College Street, New Haven CT 06520, USA; 2 Yale Cancer Center, 333 Cedar Street, New Haven CT 06520, USA; 3 Yale University School of Medicine, 333 Cedar Street, New Haven CT 06520, USA

**Keywords:** Indoor tanning, Correlates, Epidemiology, Skin cancer prevention

## Abstract

**Background:**

Despite educational and public health campaigns to convey the risks of indoor tanning, many individuals around the world continue to engage in this behavior. Few descriptive studies of indoor tanning have collected information pertaining to the lifetime history of indoor tanning, thereby limiting our ability to understand indoor tanning patterns and potentially target interventions for individuals who not only initiate, but continue to persistently engage in indoor tanning.

**Methods:**

In-person interviews elicited detailed retrospective information on lifetime history of indoor tanning among white individuals (n = 401) under age 40 seen by a dermatologist for a minor benign skin condition. These individuals were controls in a case-control study of early-onset basal cell carcinoma. Outcomes of interest included ever indoor tanning in both males and females, as well as persistent indoor tanning in females - defined as females over age 31 who tanned indoors at least once in the last three or all four of four specified age periods (ages 11-15, 16-20, 21-30 and 31 or older). Multivariate logistic regression was used to identify sociodemographic and lifestyle correlates of ever and persistent indoor tanning in females.

**Results:**

Approximately three-quarters (73.3%) of females and 38.3% of males ever tanned indoors, with a median age of initiation of 17.0 and 21.5, respectively. Among indoor tanners, 39.3% of females and 21.7% of males reported being burned while indoor tanning. Female ever indoor tanners were younger, had darker color eyes, and sunbathed more frequently than females who never tanned indoors. Using unique lifetime exposure data, 24.7% of female indoor tanners 31 and older persistently tanned indoors starting as teenagers. Female persistent indoor tanners drank significantly more alcohol, were less educated, had skin that tanned with prolonged sun exposure, and sunbathed outdoors more frequently than non-persistent tanners.

**Conclusions:**

Indoor tanning was strikingly common in this population, especially among females. Persistent indoor tanners had other high-risk behaviors (alcohol, sunbathing), suggesting that multi-faceted behavioral interventions aimed at health promotion/disease prevention may be needed in this population.

## Background

Ultraviolet (UV) radiation is recognized as the highest risk category of carcinogen by the International Agency for Research on Cancer, and UV-emitting tanning devices were elevated to this category in 2009 [1]. UV from sun exposure is the primary environmental etiologic factor for both melanoma and non-melanoma skin cancers [2-5]. While campaigns have attempted to educate people on the importance of using sunscreen, limiting exposure to strong sunlight, and preventing sunburns, indoor tanning has become an increasingly common source of UV exposure in developed countries [6,7]. Parallel to this trend have been increases in the incidence rates of both melanoma and non-melanoma skin cancer [8-12], especially among young females [13-15].

Epidemiologic evidence has identified indoor tanning as a risk factor for melanoma and squamous cell skin cancer [6,7,16], and recent results, including from our group, also identify indoor tanning as a risk factor for basal cell carcinoma (BCC) in younger people [17,18]. Additionally, etiologic studies in younger populations with a high prevalence of indoor tanning show evidence of a dose-response relationship, with higher risk observed for increasing frequency and duration of indoor tanning for both melanoma [19,20] and BCC [18].

Prevalence estimates of indoor tanning in developed countries vary widely, as anywhere from 2.8% to 47.0% of individuals report having tanned indoors in the last year, with considerable variation by age and gender [21]. Approximately 30 million people in the United States tan indoors annually, of which 2.3 million are adolescents [6]. Part of the popularity of indoor tanning may be attributed to the fact that tanned skin is portrayed as attractive and desirable in popular culture. To further complicate matters, mixed messages about sun exposure and cancer have recently emerged, with media attention focusing on the potential cancer-preventive benefits of vitamin D, which is produced when skin is exposed to UV light. Some scientists have even stated that "tanning beds may also provide some medical benefit," due to higher vitamin D levels in tanners [22]. Indoor tanning beds or booths have a widespread presence beyond indoor tanning salons, with facilities now located within gyms, beauty salons, and even people's homes. Across 116 cities in the United States, the overall mean number of commercial tanning facilities per city (mean = 41.8) was much higher than the overall mean number of two particularly common institutions, Starbucks (mean = 19) and McDonald's (mean = 29.6), in these same locations [23].

A review of 16 studies that assessed correlates of indoor tanning among various populations found that the most likely indoor tanners were females between the ages of 20 and 30 who had skin types that would become slightly burned with a moderate tan or not burned with a good tan one week after one hour exposure to sunlight [24]. An extended review of the literature that included an additional 18 studies confirmed these risk factors, but also highlighted evidence of greater differences in correlates by age and gender [21]. Among females, those who have tanned indoors were more likely to have unhealthier diets, smoke, drink alcohol, and lack correct information on the safety of indoor tanning compared to females who did not engage in indoor tanning [21]. In studies of younger populations, which were predominantly composed of females, indoor tanners were more likely to engage in other risk seeking behaviors, such as smoking, drinking and recreational drug use [25,26], and had less healthy lifestyle choices [26,27] compared with those who had never used tanning beds/booths.

Because both ever indoor tanning and persistent (long duration indoor tanning) have been associated with skin cancer risk, in this analysis, we describe the epidemiology of ever indoor tanning and persistent indoor tanning and the sociodemographic and lifestyle correlates of these behaviors among white males and females age 12-40 years in Connecticut, USA. Much of the prior descriptive research on indoor tanning has been limited to adolescents, students, or young adults, whereas our study includes a population with a broader age range. While one recent study examined a similar population, the outcome was limited to indoor tanning in the past year [28]. In contrast, we evaluated indoor tanning habits over an individual's life, with detailed information on frequency, duration, and age at initiation. Obtaining a better understanding of the prevalence and persistence of indoor tanning and associated sociodemographic and lifestyle factors may help to correctly target and tailor public health interventions to different types of indoor tanners.

## Methods

### Yale Study of Skin Health in Young People

Subjects for this analysis were controls from a case-control study of early-onset BCC, described in detail elsewhere [29]. This analysis was limited to controls, as these individuals would be more generalizable to the general population than our BCC cases. Briefly, the Yale Study of Skin Health in Young People identified BCC cases and controls with minor benign skin conditions diagnosed between July 1, 2006 and September 30, 2010 through the Yale Dermatopathology database. To be eligible, participants had to: be less than 40 years of age at the time of skin biopsy, reside in Connecticut, speak English, and either the participant (or appropriate guardian for those under age 18) had to be capable of completing all study components. Participants completed a structured in-person interview and several self-administered questionnaires. The study was approved by Yale University's Institutional Review Board and study participants (or guardians) provided appropriate written informed consent.

Potential controls were individuals diagnosed with minor benign skin conditions in the Yale Dermatopathology database during the study period. To determine a list of eligible control conditions for sampling, two dermatologists reviewed a list of all skin conditions diagnosed during a one-year period in persons under age 40 in the Yale Dermatopathology database prior to recruitment. A variety of diagnoses were determined ineligible for sampling, including skin cancers/precancers (e.g., melanoma, squamous cell carcinoma, T-cell lymphomas, actinic keratoses), potentially UV-related benign conditions (e.g., solar lentigo, atypical nevus), erythematous conditions associated with photosensitivity or aggravated by UV exposure (e.g. lupus erythematous, erythema multiforme, rosacea), dermal conditions treated with UV therapy (e.g., psoriasis), and pigment disorders (e.g., vitiligo). Randomly sampled controls were frequency matched to BCC cases on age at biopsy (5 year age groups), gender, and biopsy site (head/neck, trunk, extremity).

A total of 458 controls participated in the study, with a response rate of 60.7% among those able to be directly contacted. Any individual who self-reported a history of BCC during the interview was excluded from the control population. The 458 enrolled controls had a variety of skin conditions; cyst (16.4%), seborrheic keratosis (16.2%) and wart (11.4%) were the three most common and all other diagnoses accounted for < 10% of controls.

### Measures

During the structured interview, participants were asked about their lifetime use of indoor tanning beds/booths, including regular tanning beds/booths, high speed/high intensity tanning beds/booths, and high pressure tanning beds/booths. Participants were provided color photos of the different types of tanning beds/booths to aid in reporting. We also queried age at which participants first tanned indoors, and number of burns from indoor tanning. Across four specified age periods (ages 11-15, 16-20, 21-30 and 31 or older) frequency of use was also queried. In addition to a dichotomous ever versus never indoor tanning variable, a frequency measure of indoor tanning was calculated: total number of sessions over the four age periods. We also categorized the female ever indoor tanners who were 31 years of age or older into two groups: persistent and non-persistent tanners. Persistent indoor tanners were defined as females who tanned indoors at least once in the last three age periods (excluding the 11-15 age period) or in all four specified age periods.

In addition to sociodemographic information, several other characteristics were ascertained during the interview including: outdoor sunbathing sessions during three time periods (8-15 years old, 16-25 years old, and 26 plus years old), height, weight (age 18 and current), alcohol consumption (red and white wine, hard liquor/mixed drinks, and beer over two age periods; total number of drinks under age 25 and drinks per year ≥ age 25) and tobacco use. Participants were also asked to report their skin color (very fair, fair, light olive, dark olive, brown, very dark brown/black), eye color (grey, blue, green, hazel, brown), skin reaction to strong sunlight for the first time in summer for one hour without sunscreen (severe sun burning with blistering, painful sun burning for a few days followed by peeling, mild burning followed by some degree of tanning, turning brown without any sunburn), and skin reaction upon repeated and prolonged exposure to sunlight (very brown and deeply tanned, moderately tanned, mildly tanned due to a tendency to peel, only freckled with no suntan at all).

### Statistical analysis

This analysis is limited to the 401 (87.5%) self-identified white controls from the total pool of 458 controls. Three individuals in our analytic sample were under age 18 at the time of interview. To evaluate differences between ever indoor tanners and never indoor tanners stratified by gender, we employed the Wilcoxon Rank Sum test and chi-square test. Among the indoor tanners, we used the Wilcoxon Rank Sum test to compare distributions and the z-test to compare proportions between males and females. All variables were first entered into an unadjusted univariate logistic regression model to predict ever versus never indoor tanning. We then constructed a multivariate model using a stepwise procedure; this step was restricted to females given the limited sample size for males. Univariate and multivariate logistic regression models were also built to evaluate characteristics associated with persistent indoor tanning versus non-persistent tanning among female indoor tanners age 31 and older.

For all logistic regression analyses, reported *P *values correspond to the likelihood ratio test. For the univariate logistic regression analyses, the likelihood ratio test compares a model with the variable of interest to the null model. In the multivariate logistic regression setting, the likelihood ratio test compares the full model to the full model minus the variable of interest. Variables with a *P *value less than 0.1 were retained in the multivariate models. All analyses were conducted using R [30] and reported *P *values are two-sided.

## Results

Among the 281 females in our sample, 73.3% had tanned indoors at least once, whereas only 38.3% of the 120 males had ever tanned indoors (Table 1). The median age of the sample at the time of skin biopsy was approximately 37 years old. Both male and female indoor tanners sunbathed outdoors more frequently than those who had never tanned indoors. We did not observe any other significant differences between male indoor tanners and males who had never tanned indoors. Among females, those who had never tanned indoors had higher body mass indices (BMIs) than those who tanned indoors. Female indoor tanners were also more likely to have darker eye color and skin color, and to have skin that tanned deeply with prolonged sun exposure than females who had never tanned indoors.

**Table 1:** Selected characteristics by ever indoor tanning status stratified by gender

	Females n = 281			Males n = 120		
		**Never Indoor**			**Never Indoor**	
	**Indoor Tanners**	**Tanners**		**Indoor Tanners**	**Tanners**	
**Characteristic**	**n = 206**	**n = 75**	***P *value^a^**	**n = 46**	**n = 74**	***P *value^a^**

	Median (IQR)			Median (IQR)		

Age at skin biopsy, y	36.8 (33.2-38.4)	37.4 (34.1-39.1)	0.111	36.2 (31.2-38.4)	36.5 (31.3-38.4)	0.848
Sunbathing sessions, n	490 (268-870)	162 (51-386)	< 0.001	106 (34-458)	11 (34-458)	0.001
BMI, kg/m^2^	22.8 (20.8-26.6)	24.3 (21.5-28.6)	0.053	27.3 (25.2-35)	27.4 (24.8-30.7)	0.955
Pounds gained since age 18, lbs	15 (2-30)	16 (5-36)	0.126	25 (12.8-35)	20 (10-40)	0.672
Alcoholic drinks under age 25, n	416 (0-1664)	104 (0-1141)	0.399	2040 (0-4928)	1410 (107-3631)	0.901
Alcoholic drinks per year age 25+, n	112 (0-214)	77 (0-240)	0.688	197 (0-571)	212 (60.0-502)	0.417

	n^b ^(%)			n^b ^(%)		

Eye color, n (%)			0.035			0.705
Brown	90 (43.7)	24 (32.0)		20 (43.5)	28 (37.8)	
Hazel	41 (19.9)	14 (18.7)		8 (17.4)	11 (14.9)	
Green	17 (8.3)	15 (20.0)		1 (2.2)	5 (6.8)	
Blue	58 (28.2)	22 (29.3)		17 (37.0)	30 (40.5)	
Skin Color, n (%)			0.042			0.797
Olive	43 (20.9)	14 (18.7)		10 (21.7)	16 (21.6)	
Fair	128 (62.1)	38 (50.7)		30 (65.2)	45 (60.8)	
Very Fair	35 (17.0)	23 (30.7)		6 (13.0)	13 (17.6)	
Skin reaction with first summer sun, n (%)			0.062			0.811
Sunburn	85 (41.3)	41 (54.7)		15 (32.6)	21 (28.4)	
Tan	121 (58.7)	34 (45.3)		31 (67.4)	52 (70.3)	
Skin reaction with prolonged sun, n (%)			0.024			0.616
Deep Tan	42 (20.4)	6 (8.0)		8 (17.4)	17 (23.0)	
No/Mild/Moderate Tan	164 (79.6)	69 (92.0)		38 (82.6)	57 (77.0)	
Smoking Status, n (%)			0.214			0.190
Not Current Smoker	172 (83.5)	66 (90.4)		33 (71.7)	61 (82.4)	
Current Smoker	34 (16.5)	7 (9.6)		13 (28.3)	12 (16.2)	
Education, n (%)			0.372			0.999
Less than Bachelor's Degree	73(35.4)	21(28.0)		22(47.8)	34(45.9)	
Bachelor's Degree or more	133(64.6)	52(69.3)		24(52.2)	40(54.1)	

^a^Wilcoxon Rank Sum Test for continuous and Chi-Square test for categorical variables.

^b^May not sum to total due to missing data.

*BMI *body mass index; *IQR *interquartile range

Females started indoor tanning at a younger age than males (median 17.0 years old versus 21.5 years old, P value = < 0.001) (Table 2). Females reported significantly more indoor tanning sessions than males, with medians of 73 and 13 sessions, respectively (P value < 0.001). The maximum cumulative number of indoor tanning sessions was 4,449 among female indoor tanners and 3,860 sessions in males. We also observed that of those who tanned indoors, a significantly higher proportion of females compared to males tanned indoors between the ages of 11-15 and 16-20, but this difference was not present for the older age periods. Just under 40% of females reported ever being burned while indoor tanning, as compared to 21.7% of males (P value = < 0.001), yet the median number of burns among those who had experienced a burn from indoor tanning was not significantly different by gender (P value = 0.466). Among the 252 participants who had engaged in indoor tanning, 97.2% had used a regular indoor tanning bed/booth at least once, while only 40.5% had reported using a high speed/high intensity tanning bed/booth at least once, and 13.9% had ever used a high pressure tanning bed/booth.

**Table 2:** Tanning related characteristics by gender among indoor tanners

	Female	Male	
	Indoor Tanners	Indoor Tanners	
	n = 206	n = 46	
Characteristic	Median (IQR) or %	Median (IQR) or %	*P *value^a^
Age first tanned indoors (y)	17.0 (16.0-18.0)	21.5 (19.0-24.5)	< 0.001
Indoor tanning sessions (n)	73 (15-240)	13 (3-42)	< 0.001
Tanned indoors between ages 11-15 (%)	10.2	0	0.049
Tanned indoors between ages 16-20 (%)	81.1	45.7	< 0.001
Tanned indoors between ages 21-30 (%)	71.8	78.3	0.482
Tanned indoors between ages 31+ (%)	35.9	32.6	0.799
Ever burned from indoor tanning (%)	39.3	21.7	< 0.001
Burns from indoor tanning^b ^(n)	2 (1-4)	2 (1-3)	0.466

^a^Wilcoxon Rank Sum Test for continuous and 2-sample proportion test for percentages.

^b^Among indoor tanners who reported at least one burn.

*IQR *interquartile range

In female participants (n = 281), we identified several independent correlates of ever indoor tanning (Table 3). Females who tanned indoors were more likely to be younger than those who had never tried indoor tanning. Females with green eyes were significantly less likely to tan indoors than those with brown eyes (OR = 0.18; 95% CI = 0.07-0.50). Outdoor sunbathing during ages 8-15 and age 26 and over was also significantly positively associated with ever indoor tanning. There were suggestive associations between indoor tanning and skin reaction to prolonged sun exposure as well as pounds gained since age 18, but these did not reach statistical significance. Although skin color, skin reaction to first summer sun, and sunbathing between ages 16 to 25 were univariately associated with ever indoor tanning, these did not remain in the multivariate model after adjustment for other factors.

**Table 3:** Correlates of ever indoor tanning among females (n = 281)

		% Ever					
		Tanned	Univariate	*P *value^a^		Multivariate	*P *value^b^
Characteristic	N	Indoors	OR (95% CI)		N	OR (95% CI)	
Eye color							
Brown	114	79	1.00		101	1.00	
Hazel	55	75	0.78 (0.37 - 1.66)		51	0.76 (0.31 - 1.83)	
Green	32	53	0.30 (0.13 - 0.69)		30	0.18 (0.07 - 0.50)	
Blue/grey	80	72	0.70 (0.36 - 1.37)	0.047	74	0.72 (0.33 - 1.57)	0.008
Skin Color							
Olive	57	75	1.00				
Fair	166	77	1.10 (0.54 - 2.22)				
Very fair	58	60	0.50 (0.22 - 1.10)	0.05			
Skin reaction with first summer sun exposure							
Sunburn	126	67	1.00				
Tan	155	78	1.72 (1.01 - 2.92)	0.046			
Skin reaction with prolonged sun exposure							
Deep tan	48	88	1.00		42	1.00	
No/Mild/Moderate Tan	233	70	0.34 (0.14 - 0.84)	0.009	214	0.37 (0.12 - 1.18)	0.07
BMI (kg/m^2^)	280	-	0.97 (0.92 - 1.01)	0.117			
Pounds Gained since age 18 (per 10 lbs)	278	-	0.81 (0.66 - 0.99)	0.037	256	0.80 (0.63 -1.02)	0.063
Alcoholic drinks under age 25 (per 300 drinks)	275	-	1.01 (0.98 - 1.05)	0.454			
Alcoholic drinks/year over age 25 (per 100 drinks)	262	-	1.00 (0.90 - 1.11)	0.988			
Smoking Status							
Not Current Smoker	238	72	1.00				
Current Smoker	41	83	1.86 (0.79 - 4.41)	0.136			
Education							
Less than Bachelor's Degree	94	78	1.00				
Bachelor's Degree or more	185	72	0.74 (0.41 - 1.32)	0.296			
Sunbathing session ages 8-15 (per 50 sessions)	279	-	1.25 (1.13 - 1.39)	< 0.001	256	1.26 (1.11 - 1.42)	< 0.001
Sunbathing session ages 16-25 (per 50 sessions)	279	-	1.18 (1.09 - 1.29)	< 0.001			
Sunbathing session ages 26+ (per 50 sessions)	257	-	1.29 (1.12 - 1.49)	< 0.001	256	1.23 (1.05 - 1.44)	0.003
Age at skin biopsy	281	-	0.98 (0.93 - 1.04)	0.549	256	0.90 (0.81 - 0.99)	0.037

^a^P values are from likelihood ratio tests comparing a model with the variable of interest to the null model.

^b^P values are from likelihood ratio tests comparing the full model to the full model minus the variable of interest.

*BMI *body mass index; *CI *confidence interval; *OR *odds ratio

Among the 170 females age 31 and over who had tanned indoors at least once, a total of 42 (24.7%) were defined as persistent indoor tanners. All variables that were univariately associated with persistent indoor tanning remained significant correlates in the multivariate model (Table 4). Persistent indoor tanners reported significantly more alcohol consumption before age 25 (per an additional 300 drinks under age 25 OR = 1.05; 95% CI = 1.01-1.09) than non-persistent tanners, with a median of 634 total drinks under age 25 versus 274 total drinks under age 25, respectively. Females with skin that tanned with prolonged sun exposure were also much more likely to engage in persistent indoor tanning compared to the occasional indoor tanner. Education level was inversely associated with persistent indoor tanning, with females completing at least a bachelor's degree being 70% less likely to persistently indoor tan than those with less education (OR = 0.29; 95% CI = 0.14, 0.63). In addition, persistent indoor tanning among females in our sample was positively associated with outdoor sunbathing sessions between the ages of 16 and 25 (per 50 sessions OR = 1.10; 95% CI = 1.00, 1.21), with a median number of sunbathing sessions of 360 for persistent indoor tanners and 240 for non-persistent indoor tanners during this age period.

**Table 4:** Correlates of persistent indoor tanning among female indoor tanners age 31 and over (n = 170)

		% Persistently					
		Tanned	Univariate			Multivariate	
Characteristic	N	Indoors	OR (95% CI)	*P *value^a^	N	OR (95% CI)	*P *value^b^
Eye color							
Brown	71	28	1.00	0.431			
Hazel	36	28	0.98 (0.40 - 2.40)				
Green	14	29	1.02 (0.29 - 3.63)				
Blue/grey	49	16	0.50 (0.20 - 1.25)				
Skin color							
Olive	33	27	1.00	0.902			
Fair	106	24	0.82 (0.34 2.0)				
Very fair	31	26	0.93 (0.31 - 2.82)				
Skin reaction with first summer sun exposure							
Sunburn	76	18	1.00	0.085			
Tan	94	30	1.88 (0.91 - 3.90)				
Skin reaction with prolonged sun exposure							
Deep Tan	35	40	1.00	0.023	35	1.00	0.031
No/Mild/Moderate Tan	135	21	0.39 (0.18 - 0.87)		135	0.39 (0.16 - 0.91)	
BMI (kg/m^2^)	170	-	1.02 (0.96 - 1.08)	0.535			
Pounds Gained since age 18 (per 10 lbs)	170	-	1.14 (0.86 - 1.49)	0.364			
Alcoholic drinks under age 25 (per 300 drinks)	170	-	1.05 (1.01 - 1.09)	0.015	170	1.05 (1.01 -1.09)	0.015
Alcoholic drinks/year over age 25 (per 100 drinks)	170	-	1.09 (0.96 - 1.23)	0.156			
Smoking Status							
Not Current Smoker	144	23	1.00	0.217			
Current Smoker	26	35	1.78 (0.73 - 4.37)				
Education							
Less than Bachelor's Degree	57	39	1.00	0.003	57	1.00	0.002
Bachelor's Degree or more	113	18	0.34 (0.17 - 0.70)		113	0.29 (0.14 -0.63)	
Sunbathing session ages 8-15 (per 50 sessions)	170	-	1.09 (0.99 - 1.21)	0.087			
Sunbathing session ages 16-25 (per 50 sessions)	170	-	1.10 (1.01 - 1.20)	0.033	170	1.10 (1.00 -1.21)	0.044
Sunbathing session ages 26+ (per 50 sessions)	169	-	1.09 (0.99 - 1.20)	0.086			
Age at skin biopsy	170	-	0.93 (0.80 - 1.08)	0.344			

^a^*P *values are from likelihood ratio tests comparing a model with the variable of interest to the null model.

^b^*P *values are from likelihood ratio tests comparing the full model to the full model minus the variable of interest.

*BMI *body mass index; *CI *confidence interval; *OR *odds ratio

## Discussion

In this study, we sought to better characterize individuals under age 40 who engaged in indoor tanning. Indoor tanning was strikingly common in our population, with approximately three-quarters of females and just over one-third of males having tanned indoors at least once before age 40. Our finding that females were more likely than males to use tanning beds is well supported in the literature [21,24,31]. Our data on indoor tanning across specific age periods indicated that both males and females were most likely to have engaged in this activity under age 30, which is also in line with existing research [21,24,31].

With unique comprehensive lifetime indoor tanning data, we found that one quarter of female indoor tanners 31 or older were persistent tanners, as they had engaged in indoor tanning throughout the queried age periods. The persistent female indoor tanners tended to be less educated, drank more alcohol, and sunbathed outdoors more frequently than other females who only occasionally tanned indoors. Among these older females, the correlates of persistent indoor tanning were similar to findings among college-age and adolescent females where indoor tanning has been positively associated with both risk taking [25,26] and unhealthy lifestyle [26,27] behaviors. Since indoor tanning is less common in males there is less descriptive research in this population, yet a recent study in adolescent males found that indoor tanning correlated with binge drinking, unhealthy weight control practices, and steroid use [32]. The clustering of addictive behaviors, such as smoking and drinking, with indoor tanning may reflect more general risk taking behavior, although it may also correlate with an addictive tendency, since some have posited that indoor tanning may be a biologic dependency [33-36]. Additional research on addiction in relation to indoor tanning is needed because interventions aimed at those who persistently tan indoors may need to focus on addiction rather than skin health or cancer prevention messages.

Pigment characteristics such as eye color, skin color, and skin reaction to UV exposure are commonly used to identify individuals at higher risk of skin cancer [4,16,29]. Overall, we found that people with lighter pigment traits who would be more likely to burn with UV exposure were less likely to engage in indoor tanning compared to those with darker phenotypes. Nonetheless, within the most at-risk phenotypes, there was still a large proportion of participants, especially females, engaging in indoor tanning. For example, among the 14 females who said that prolonged sun exposure would yield no tan and just freckling, approximately 30% had tanned indoors at least once in their lifetime. Similarly, among females who reported they would experience at least some burning upon one hour exposure to the summer sun, 68% had tanned indoors at least once.

Strengths of our study include the detailed lifetime indoor tanning history that enabled us to determine not only ever versus never use of indoor tanning beds/booths, but also frequency as well as persistence over time to evaluate potential differences between different types of indoor tanners. From the detailed in-person interview, we also had a wide range of sociodemographic and lifestyle variables to evaluate as potential correlates. A limitation to our study is that our participants were controls with a benign skin biopsy from a case-control study on BCC. It could be argued that this group of participants may be more aware of their skin health or overall health than the general population, as they were seen by a dermatologist. It is unclear what effect, if any, this choice of a study population could have on the applicability of our findings to the general population. If our controls were more aware of their skin health than other persons under age 40 in Connecticut, the true use of indoor tanning in this age group could be higher than what we observed. Alternatively, our population might be enriched in persons focused on their appearance and therefore might be more likely to use indoor tanning. While our control group might affect our prevalence estimates, this has less of an impact on the correlates of tanning. Another limitation to this study was the self-reported nature of all of the measures of interest. Our method of data collection relied on participants' willingness to give complete and true answers and also on their ability to accurately recall behavior at different points in their lives.

Age of intense UV exposure may be particularly relevant in relation to skin cancer, as evidence suggests freckling as a child (due to genetic factors and sun exposure) and sunburns early in life are particularly associated with risk of skin malignancies [2-4]. In our population, females had a much earlier age of first use of indoor tanning than males. The incidence of both melanoma and non-melanoma skin cancer is increasing [8-12] with a marked increase among young people, especially females [13-15]. Indoor tanning may underlie, at least partially, this temporal trend. In addition, with several recent studies finding positive dose-response effects for skin cancer with increasing indoor tanning [18-20], frequency and duration of exposure in addition to ever versus never use are important factors to consider for risk. In our own early-onset BCC case-control study, ever indoor tanning was associated with a two-fold (OR = 2.14 95% CI = 1.31-3.47) increased risk of BCC compared to never indoor tanning among all females [18], yet in females over age 31, the risk associated with persistent indoor tanning versus never indoor tanning was even stronger (OR = 2.76, 95% CI = 1.30-5.86) (data not shown).

While individual-level interventions are one avenue for reducing use of tanning beds/booths, legislation has also been enacted to curtail youth utilization of indoor tanning. A telephone survey of indoor tanning facilities in 116 cities in the United States found that businesses in states with youth access laws to tanning beds were significantly more likely to state they would require written permission from a parent for a minor to use the facility [37]. However, not all research supports policy level changes as a sole means to curtail youth access. A survey of adolescents in the 100 most populous cities in the United States did not find an association between indoor tanning and residing in a state with youth access laws [38], and studies which employed face-to-face interactions between an indoor tanning facility and a potential youth client found a lack of compliance with parental consent policies [39,40].

## Conclusions

Growing evidence of an increased risk of skin cancer associated with indoor tanning (both ever and persistent) suggests that targeted interventions could have a substantial impact on primary prevention of these all-too-common and potentially lethal malignancies. In addition, given the young age at initiation, high prevalence of use, and other correlated risk-taking behaviors (outdoor sunbathing, alcohol), young females who tan indoors are an ideal group to target with not only indoor tanning reduction/cessation interventions, but also other health promotion messages, including those related to outdoor UV protection and alcohol consumption.

## Abbreviations

BCC: basal cell carcinoma; BMI: body mass index; CI: confidence interval; IQR: interquartile range; OR: odds ratio; UV: ultraviolet.

## Competing interests

The authors declare that they have no competing interests.

## Authors' contributions

KL conducted the statistical analyses and LMF led the writing; both interpreted the analyses. AMM supervised the analyses. STM, AEB, DL, and BC conceived of, obtained funding for, and oversaw the Yale Study of Skin Health. All authors assisted in interpreting the results, critically edited the manuscript, and contributed substantially to the final version. All authors read and approved the final manuscript.

## Pre-publication history

The pre-publication history for this paper can be accessed here:

http://www.biomedcentral.com/1471-2458/12/118/prepub
